# Clinical outcomes of maitland mobilization in patients with Myofascial Chronic Neck Pain: A randomized controlled trial

**DOI:** 10.12669/pjms.37.4.4220

**Published:** 2021

**Authors:** Maryam Shabbir, Naveed Arshad, Anam Naz, Nadia Saleem

**Affiliations:** 1 Dr. Maryam Shabbir, Ph.D. (Rehab) scholar, Associate Professor, Riphah International University, Lahore Pakistan; 2 Dr. Naveed Arshad, M.Phil. (Rehab), Assistant Professor, Islamabad Medical and Dental College, Islamabad Pakistan; 3 Dr. Anam Naz, MS (Rehab), Senior Lecturer, The University of Lahore, Lahore Pakistan; 4 Dr. Nadia Saleem, MS (Rehab), Assistant Professor, Bakhtawar Amin College of Rehabilitation Sciences, Multan Pakistan

**Keywords:** Myofascial pain syndromes, Manual mobilization, Neck pain, Therapeutics exercises, Visual analog scale

## Abstract

**Background and Objective:**

Myofascial neck pain is a common musculoskeletal problem caused by presence of trigger points and local and referred pain patterns. Chronic neck pain is responsible for the involvement of joints, ligaments, fascia and connective tissue as well. The objective of this study was to assess the effect of Maitland mobilization in patients with myofascial chronic neck pain.

**Methods:**

In this randomized, placebo treatment-controlled trial, 30 patients consecutively aged 25-45 years meeting inclusion criteria were isolated into two groups. The study group was treated with Maitland mobilization consistently for eight weeks while the control group got placebo treatment for a similar timeframe. Visual analog Scale (VAS), Neck disability index (NDI) and cervical range of motion (ROM) questionnaire was filled by patients before, intermediate and after the intervention to evaluate the severity of pain, functional ability and range of motion.

**Results:**

Following eight weeks of treatment, when compared the post treatment effects of both groups, the significance value for VAS was 0.008, for NDI p=0.030, for Flexion p=0.573, for extension p=0.001, for right rotation p<0.001, for left rotation p=0.002, for right and left side bending p<0.001.

**Conclusion:**

The study concluded that Maitland mobilization grades (I-IV) are effective in reducing pain and improving functional level of NDI scale and the ranges of cervical spine in patients with myofascial chronic neck pain.

## INTRODUCTION

Neck pain is a most common issue which has manifold impacts on economy of society.^1^ Syndrome in which triggers points are responsible for local and referred pain in body is called myofascial pain syndrome. Sometimes it is labeled as specific disorder and soft tissue pain. Most common symptoms of above discussed is dull, deep and localized pain in muscle groups. other symptoms are fatigue, tenderness, stiffness, decreased participation restriction and activity limitation and inability to perform coordinated movements. This pain is equally prevalent in both genders. It is debilitating issue which needs more attention all over the world.^2^,^3^ Patients with neck pain syndrome face difficulty to perform activities of daily life with involvement of weakness of neck muscles that plays an important role in deep bending and increased forward head posture deformities, impairments of balance and coordination and impaired proprioception sense.^4^-^6^ Physical therapy interventions such as electrotherapy, manual therapy and exercise therapy.^7^

Mobilization grades are used to reduce pain and increase range of motion in patients with different disabilities. Maitland mobilization is one of the most common approach that is used by physical therapist.^8^,^9^ The technique in which to and for movements or oscillations are applied to the affected areas in order to improve range of motion and stiffness and also reduced pain as well that is proven by many researchers and clinical trials. These techniques are applied to spinal and vertebral joints of the body. In Grade-I, mobilization is applied with small amplitude with low resistance and its application is useful in treating pathological conditions. While in Grade-II, mobilization with wider amplitude below pain range in patients with different pathologies. Use of grade I and II are used when resistance is applied before the limit of pain. In Grade-III and IV, to and fro movements are applied on joints to remove stiffness and contractures and improve efficiency of movements. Velocity in Grade IV is high as compared to Grade-III, II and I. while grade V is a thrust with high velocity that is used in manipulation. Maitland technique is also a stretching technique that plays a significant role in protection of muscle spasm 10,11

When patient is capable to perform more than sixty percent of normal range of movements due to pain then techniques are applied to increase range and to reduce pain in affected joints and muscles.^11^ Maitland argues that the equivalent pain response “is nearly always found with the non-physiological movement rather than the physiological movement”.

So many studies have been conducted on Maitland mobilization concept to reducing pain and improving range of motion (ROM). Some studies used Maitland mobilization Grades I & II only and some used grades III & IV only. The rationale behind this study was to evaluate the theory that Maitland mobilization may reduce pain symptoms, neck disability and improve cervical range of motion in myofascial chronic neck pain. So, this study provided us with fresh first hand and local evidence of Maitland mobilization concept (Grades I-IV).

## METHODS

This interventional randomized controlled (Registration number: ClinicalTrials.gov Identifier: NCT04660292) preliminary was performed from August to September 2020 in the Riphah Rehabilitation Center, Lahore. After informed written consent thirty (30) consecutive patients (sample size was calculated from Epitool, whereas Mean 1=53.9, Mean 2=45.8, Variance=5, Confidence Interval=0.95, Power=0.8, Tails=2)^12^ meeting the inclusion criteria were enrolled. Analysis was set up based on clinical assessment and X-rays anteroposterior (AP) and lateral neck view. Patients age between 25-45 years old, having bilateral neck pain and MTrPs in upper trapezius and levator scapulae muscles for at least three months with a pain intensity of at least 2 cm on a 10 cm visual analogue scale (VAS) and greater than or equal to 15 points on neck disability index (NDI); were included in this study. Traumatic injuries (e.g., contusion, fracture, and whiplash injury; neurologic disorders (e.g., trigeminal neuralgia or occipital neuralgia); concomitant medical diagnosis of any primary headache (tension type or migraine); and clinical diagnosis of cervical radiculopathy or myelopathy were excluded from the study. Patients with Covid-19 positive, findings on PCR were also excluded from the study.

Forty patients were contacted out of which 30 patients were met the inclusion criteria and recruited by non-probability consecutive sampling. Patients were divided into two groups, Maitland mobilization (study group) and conventional physiotherapy (placebo group) by randomization sequence computer-generated numbers by a biostatistician and allocation was sealed in opaque envelopes to ensure concealment. If patients were dropped out due to Covid-19 pandemic, the new patients were included according to above criteria. Patients were examined by a physiotherapist and were allotted to study or control group (n=15 each). Patients were thoroughly examined to rule out any pathology to fulfill inclusion criteria. Cervical ranges were measured through goniometer. Palpation technique was used to check the presence of trigger points in trapezius and levator scapulae muscle (both techniques were assessed in sitting position). A predesigned Performa was used to collect demographic data as age and gender of patient.

The study group was treated by manual therapist with Maitland mobilization and manipulation techniques including postero-anterior Maitland mobilization (Grade I & II) for C1-C2, Maitland lateral PA glide (Grade-III & IV) for C3-C6 and Maitland mobilization with thrust (Grade-IV) for cervicothoracic junction. Frequency of mobilization was two days a week for eight weeks. While intensity of mobilization was Grade-3 and 4 based on the Maitland concept.^8^ Time of oscillations was two or three oscillations in a second for one to two minutes. While placebo treatment with conventional physiotherapy (active exercises-10 repetitions in all direction in pain free range, isometrics 5-10 seconds brief but maximum contraction each held for 5-16 seconds for flexors, extensors, side flexors and rotators)^12^ without gliding, oscillations and thrust were recommended for the control group. Both groups were treated with baseline treatment including TENS 10 minutes and moist hot packs in sitting position for 15 minutes on cervical region in with head resting on table with a pillow.

All numerical data was subjected to statistical analysis with Mann Whitney U Test. Kolmogorov–Smirnov and Shapiro–Wilk test was used for normality data between the groups. P-value ≤ 0.05 was considered significant.

### Ethical Approval

(Ref: RCR & AHS/REC.PhD/333, Dated: December 22, 2019)

## RESULTS

Thirty patients meeting the inclusion criteria were considered. After drop out due to Covid-19 pandemic, two new patients in study group and one new patient in placebo group were again added by selecting through the above procedure. Kolmogorov Smirnov and Shapiro-Wilk were applied as tests for normality and there found all the data normative (p ≥ 0.05) throughout the assessment. Out of 30 patients, 12 (40%) male and 18 (60%) were female. The mean age of the study group was 34.87±6.70 years, while the mean age of placebo group was 36.13±6.50 years, the minimum age was 25 years while maximum age was 45 years.

In our study Table-I shows, primary symptoms were pain and neck disability. As indicated by the Mann Whitney U Test, no distinction was seen in pain between the two groups (p=1.000). 3 were the mean score in each group. However, a significant difference was seen between the two groups after treatment (p ≤ 0.008).

**Table-I:** Comparison of pre- and post-intervention for pain between groups; n = 30.

Visual Analogue Scale (VAS)	Study group	Placebo	U*	P-value
	
	Mean±SD	Mean±SD		
At baseline week 0	2.80±0.41	2.80±0.41	112.50	1.000
At week 4	2.00±0.38	2.13±0.35	98.50	0.325
End of treatment week 8	0.87±0.35	1.33±0.49	65.00	0.008

* : Mann Whitney U test.

As regards neck disability index, no critical difference between the two groups was seen at the start of study (p=0.567). Table-II. However statistically significant difference was observed after treatment (p ≤ 0.030).

**Table-II:** Comparison of pre- and post-intervention for neck disability (NDI) between groups; n = 30.

Neck Disability Index (NDI)	Study group	Placebo	U*	P-value
	
	Mean±SD	Mean±SD		
At baseline week 0	2.87±0.64	2.73±0.59	100.50	0.567
At week 4	2.20±0.56	2.27±0.70	104.00	0.690
End of treatment week 8	1.13±0.64	1.73±0.70	65.00	0.030

* : Mann Whitney U test

The acquired outcomes were comparable for cervical range of motion, demonstrating a statistically significant difference in cervical extension simply after the treatment period (p ≤ 0.003) while non-significant in cervical flexion range of motion after treatment (p ≥ 0.573).

Cervical right and left rotation were measured from beginning and end of treatment course (Fig.2). The mean score between the two groups before the treatment was non-significant (p=0.062 versus 0.112, respectively), yet a progressively significant difference was seen after the treatment course (p ≤ 0.001 versus p ≤ 0.002, respectively).

**Fig.1:**
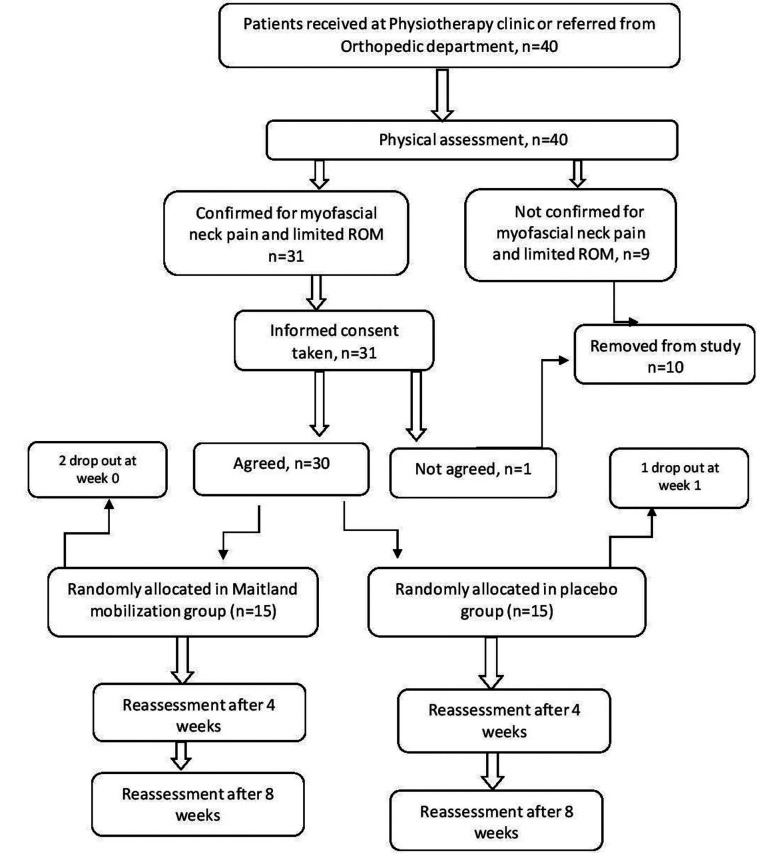
Methodology flow chart.

**Fig.2:**
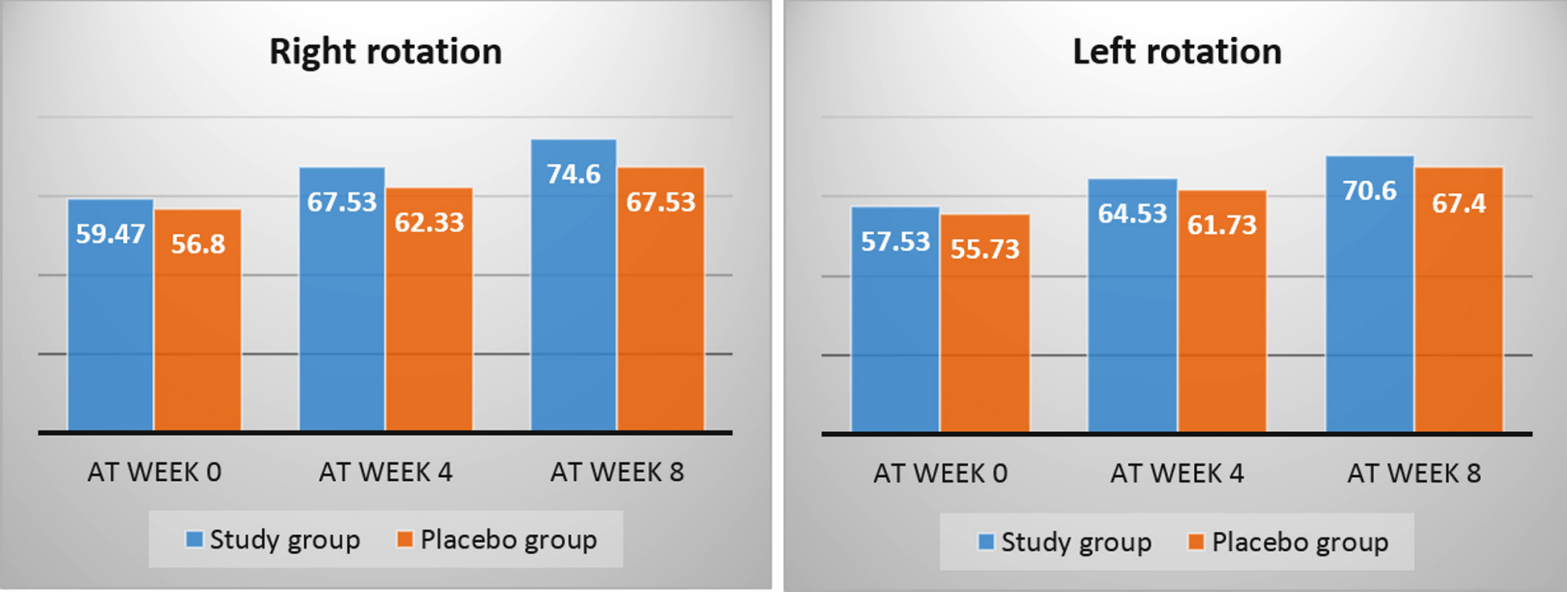
Comparison of pre- and post-intervention for range of motion (ROM) between groups.

**Fig.3:**
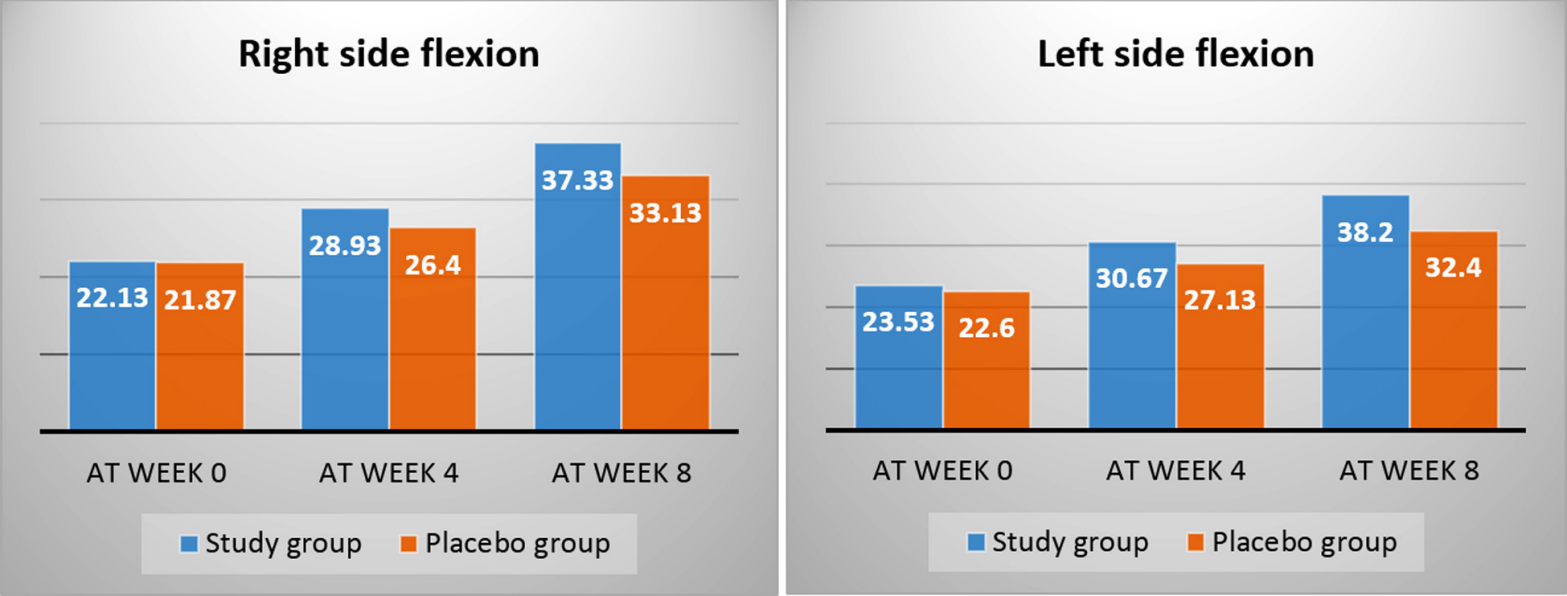
Comparison of pre- and post-intervention for range of motion (ROM) between groups.

Additionally, cervical right-side flexion and left side flexion was measured from beginning and end of treatment course (Fig.3). The mean score between the two groups before the treatment was non-significant (p=0.818 versus 0.548, respectively), at the end of treatment course a progressively significant difference was seen (p ≤ 0.001 versus p ≤ 0.001, respectively).

## DISCUSSION

Comparison was made between the impacts of Maitland mobilization in addition to physical therapy interventions in patients with myofascial chronic neck pain. Results of above-mentioned study showed reduced pain and increased range of motion and improved neck disability index (NDI) with Maitland therapy when it was compared with control group. Although, participants in both groups showed decreased in pain over time. But the pain reduction in Maitland group was because of neuro physiological, sympathetic and psychological effects of mobilization.^13^ The marked difference was seen among the Maitland mobilization and placebo groups except flexion, both clinically and statistically. Improvement in neck pain is clearly visible in segmental movement. At the start, and end of each treatment session of Maitland’s mobilization, outcome of segmental mobility was measured. According to the level of these changes the treatment was then adapted (3^rd^ grade to 4^th^ grade of Maitland concept).

It is noted that changes in sessions are valid and seen long-lasting changes with immediate effect also.^14^ Ganesh GS, et al.^15^ applied grade one and grade two of Maitland concept in their study. Mechanisms by which Maitland mobilization improved range of motion are mechanical and neuro physiological. Mechanical effects played significant role in permanent and temporary changes in length of connective tissues like joint capsule of the zygapophyseal joints, muscles and ligaments. While neurophysiologic mechanism could improve mobility in response to application of posterior anterior forces by improving the perception of pain.^16^

Current study results showed Maitland mobilization and manipulation was valuable in improving outcomes. Many studies have explained correlation of neck muscle atrophy and neck pain. Decrease in muscle strength is caused by inhibition effect of pain and muscular changes.^17^ Deep muscle weakness affect spinal posture and eventually lead to postural abnormalities and caused pain and muscle weakness.^18^ Maitland mobilization played an important role to improve pain by enhancing muscular strength.

Pain free ROM is important for normal movement. Low score of NDI in all subjects are due to reduction of pain and increased in range. Moses MJ, et al.^19^ demonstrated that NDI is specific to change in values and showed correlation with visual analogue scale.

In current study, at first week, fourth week and eighth week mean difference between values of Neck Disability Index, Visual Analogue Scale and range of motion of spine such as flexion, right and left side flexion, extension and rotation of all sides was concluded. Mean and standard deviation of above-mentioned data can be seen in Table-I, II & III in results at three intervals when both groups were compared for treatment efficiency. In the last treatment week, significance values were seen in VAS such as 0.008, for NDI p= 0.030, for side flexion, extension and rotation it was significant. For flexion it was non-significant p ≥ 0.573. Hence, pre, mid and post treatment showed improvement when both groups compared and significance difference was only seen at end of treatment session in VAS, NDI and range of motions except one movement which was flexion.

**Table-III:** Comparison of pre- and post-intervention for range of motion (ROM) between groups.

Cervical Range of Motion (ROM)		Study group	Placebo	U*	P-value

		Mean±SD	Mean±SD		
Flexion (degree)	At baseline week 0	24.67±4.34	24.13±4.03	105.50	0.771
	At week 4	31.47±4.10	30.13±3.93	88.50	0.317
	End of treatment week 8	38.60±3.16	38.00±2.93	99.00	0.573
Extension (degree)	At baseline week 0	51.47±4.05	48.00±4.34	62.00	0.036
	At week 4	57.73±3.43	53.33±3.66	57.50	0.022
	End of treatment week 8	64.00±2.98	60.07±3.11	41.00	0.003

### Limitation & recommendation of the study

This study was conducted on patients with trigger points in trapezius and levator scapulae. We did not look for the effects of distant trigger points or the latent trigger points that did not showed jump sign. In addition as a baseline treatment consisting of TENS and moist heat was also given to both groups throughout the study period which masks the effects of single technique because it had its own analgesic effects. Study was not conducted for a long time period to check the reoccurrence of MTrPs in the selected subjects which can be observed to rule out the long-term beneficial therapy. Moreover, eight weeks is not a prolonged follow up. There should be a follow up for long duration to check the reoccurrence of trigger points and associated complaints.

## CONCLUSION

Maitland mobilization technique (grades I-IV) is effective in reducing pain and improving functional level of NDI scale and the ranges of cervical spine in patients with myofascial chronic neck pain. There was statistical difference between the groups to favor Maitland techniques.

### Authors’ Contribution:

**MS:** Provided concept/research design and did data collection.

**NA and AN:** Did statistical analysis and manuscript writing.

**MS & NA:** Did editing of manuscript and project management.

**NS:** Did critical revision of the manuscript for important intellectual content.

**NA & MS:** Takes the responsibility and is accountable for all aspects of the work in ensuring that questions related to the accuracy or integrity of any part of the work are appropriately investigated and resolved.
